# Establishment and characterization study of ovine mammary organoids

**DOI:** 10.1186/s12917-025-04657-4

**Published:** 2025-03-19

**Authors:** Rui Feng, Sijia Ma, Ruixue Bai, Yijing Zhu, Sarengele, Jingru Ning, Quanzhong Xu, Chunwei Wang, Lequn Wang, Chao Bian, Zhong Zheng, Pengfei Shou, Liguo Zhang, Xiaohu Su

**Affiliations:** 1 State Key Laboratory of Reproductive Regulation and Breeding of Grassland Livestock, School of Life Sciences, Inner Mongolia University, Hohhot, Inner Mongolia Autonomous Region China; 2 Ulanqab Animal Husbandry Workstation, Ulanqab Agriculture and Animal Husbandry Bureau, Ulanqab, 012000 Inner Mongolia Autonomous Region PR China; 3 Tumor radiotherapy department, Inner Mongolia People’s Hospital, Hohhot, 010017 Inner Mongolia Autonomous Region China; 4 Animal health quarantine and inspection Center, Ulanqab Agriculture and Animal Husbandry Bureau, Ulanqab, 012000 Inner Mongolia Autonomous Region PR China

**Keywords:** Mammary organoids, Sheep, Characterization, Transfection

## Abstract

The study of ovine lactation and mastitis is limited by the lack of physiologically relevant in vitro models. Compared to mammary epithelial cells, mammary gland organoids (MaOs) offer a better model for studying lactation, as they more accurately replicate the tissue structure and interactions of cells in vivo. Here, we optimize a protocol for the isolation, culture, characterization, and transfection of ovine MaOs derived from ewe mammary tissue. Results: We compared different enzymatic digestion methods and found that type IV collagenase digestion for 60 min yielded the highest number of MaOs compared to a mixed enzyme solution of type II collagenase and hyaluronidase. Culture medium optimization revealed that medium B supported superior growth, with increased budding structures, a higher number of MaOs over time, and a greater proportion of EdU-positive proliferating cells compared to media A and C. Cell-type characterization confirmed the presence of both luminal and myoepithelial cells, as shown by RT-PCR analysis of *CK14*, *CK18*, and *CK7* expression, and further validated by immunofluorescence staining for CK14 and CK18. MaOs in medium B exhibited significantly higher mRNA expression levels of milk lipid metabolism-related genes (*XDH*, *FABP3*, *SREBP1*), lactose metabolism-related genes (*GLUT1*, *GLUT4*), and milk protein synthesis genes (*EIF4E*, *CSN2*) at multiple time points. In all media, *XDH*, *FABP3*, *SREBP1*, *GLUT1*, *GLUT4*, *EIF4E*, and *CSN2* mRNA expression peaked at Day 7 before declining. Additionally, at Day 7, supernatant analysis confirmed that triglyceride, lactose, and CSN2 concentrations were significantly elevated in medium B compared to media A and C. Finally, we demonstrated that the ovine MaOs could be transfected using electroporation with 27.3% becoming GFP-positive. Conclusions: This study establishes a protocol for the isolation, culture, characterization, and genetic manipulation of ovine MaOs. These organoids serve as a physiologically relevant model for studying the regulatory mechanisms of lactation and mastitis in sheep, providing a tool for future research in veterinary and agricultural sciences.

**Supplementary Information:**

The online version contains supplementary material available at 10.1186/s12917-025-04657-4.

## Introduction

The protein and fat contents of ovine milk are higher than bovine and caprine [1]. Its products can supply fatty acids, calcium, phosphorus, iron and magnesium for human [2]. In addition, the ovine milk also contains many biologically active substances, such as antibacterial, immunomodulatory and antioxidant [3]. However, the exploratory research of ovine lactation regulatory mechanism is still insufficient due to the lack of appropriate in vitro model.

Previous in vitro model of biological problems research mainly was cell lines. Nevertheless, many biological processes involve interactions of different cell types and monolayer cells are difficult to reveal the real biological processes in vivo due to the lack of tissue architecture and complexity. For example, once removed from their native tissue environment and cultured in vitro, the mammary luminal epithelial cells would lose the interaction with the microenvironment further leads to changes of phenotype and morphology, even loss of polarity [4]. Recent advances of the organoid have revolutionized the in vitro culture models for biologic research through three-dimensional (3D) models creation to recapitulate the cellular heterogeneity, structure, and functions of primary tissues [5]. At present, the mouse and human mammary organoids (MaOs) were widely studied, of which were established from fragments of the mammary cavity epithelium [6, 7]. The availability of MaOs from livestock is important from the perspective of providing a physiologically relevant model for mammary development and lactation traits mechanism exploratory research. Until now, the culture systems of bovine, porcine, canine and equine MaOs have been established [8–12]. However, the culture system of ovine MaO is still lacking. It may limit the dairy ovine lactation traits mechanism exploratory and mastitis study.

For lack of known markers to identify progenitor/stem cells in the mammary gland in non-traditional model species, the ovine sorting of mammary stem cells is difficult. And the culture system of ovine mammary stem cells is not effective. So the utilization of mammary tissue fragments to isolate the ovine MaOs would be a feasible method. Here, the isolation condition of the ovine MaOs was first explored. Then the culture medium was optimized based on the proliferation capacity analyzing. Further, the cell types and lactational function were characterized. Finally, the transfection system of ovine MaOs was established to facilitate subsequent applications. Together, this study firstly reports the establishment and characterization of ovine MaOs, and would provide a new tool for the study of ovine regulatory mechanism of lactation traits and mastitis.

## Materials and methods

### Ethics statement

This study received the approval from the Inner Mongolia University Research Ethics Committee (approval number: 2021002). All experiments were performed according to Chinese laws and institutional guidelines.

### Mammary tissue collection and digestion

Mammary tissues were collected from mid-lactating ewes (Small-Tailed Han sheep, 2 ∼ 3 years old, *n* = 3) of local slaughter house. The collected mammary tissues were washed with 0.9% saline solution (200 U/mL penicillin (Yeasen, China) and 200 µg/mL streptomycin (Yeasen, China) were added) until the blood and milk were removed. Then redundant adipose and connective tissues were cut out and washed with phosphate-buffered solution (PBS, 200 U/mL penicillin and 200 µg/mL streptomycin were added) for 3 times. After sterilized with 75% ethanol for 2 min, the tissues were minced into 1 ~ 3 mm^3^ pieces. The minced tissues were digested with 1.5 mg/mL type IV collagenase (Solarbio, China) (Digestion A) or 1.5 mg/mL type II collagenase (Solarbio, China) plus 1.0 mg/mL hyaluronidase (Solarbio, China) (Digestion B) for 45 ∼ 90 min with rocking at 37℃. After incubation, tissues were filtered with 120 mesh cell sieve and resuspended in the DMEM (Yeasen, China) with 2.5% BSA (MedChem Express, USA). Then the mixture was centrifugated at 1500 RPM for 15 min at room temperature. The MaOs were contained at the middle layer.

### MaOs plating and culturing

Firstly, the middle layer was aspirated and diluted with the DMEM (200 U/mL penicillin and 200 µg/mL streptomycin were added). Then diluted mixture was centrifugated at 1500 RPM for 10 min at room temperature. And then the differential centrifugation (1500 RPM for 3 ∼ 4 s, 3 ∼ 4 times) was used to remove residual enzymes and single cells. Finally, the precipitation was resuspended with the DMEM (200 U/mL penicillin and 200 µg/mL streptomycin were added). The MaOs were spheroids with a smooth edge. The density was calculated as MaOs number/1 g mammary tissue. After centrifugation, the MaOs were resuspended with pre-chilled Matrigel (Yeasen, China) at the concentration of 200/mL and plated into wells. The culture plates (Nest, China) were incubated at 37℃ for 10 ∼ 20 min to coagulation. Then different mediums were added and cultured at 37℃, 5% CO_2_ and saturated humidity. The components of three mediums were followed: Medium A: DMEM + 0.1% BSA (Sigma, USA) + 20 ng/mL EGF (Sigma, USA) + 2 nM L-Gln (Sigma, USA) + 1X ITS (Yeasen, China) + 100 ng/mL FGF2 (Sigma, USA) + 1 µg/mL Hydrocortisone (Sigma, USA) + 4 µg/mL heparin (Sigma, USA) + 100 U/mL penicillin + 100 µg/mL streptomycin; Medium B: 1 µg/mL PRL was added based on Medium A; Medium C: the BSA was replaced with 10% FBS (Yeasen, China) of Medium A. The medium was changed every 2 ∼ 3 days.

### Morphology and proliferation capacity assessments

The morphology and proliferation capacity of cultured MaOs at D3, D7, D14 and D21 were assessed. The bright-field images of MaOs were obtained using a Zeiss Axio Observer inverted microscope (Zeiss, Germany). The area of organoids was calculated by the imaging software. Ten randomly fields per well were selected and three wells per individual sheep of each condition were used to determine the number and area of organoids. The calculation of organoid formation efficiency as MaOs number of D3/MaOs number of plated (D0) × 100%.

The EdU staining was performed using the SFClick™ EdU-488 Proliferation assay Kit (SEVEN, China) according to the manufacturer’s instructions. At Day 7, MaOs were supplemented with 10 µM EdU for 4 h at 5% CO_2_ and 37℃. Than digested with 0.25% trypsin for 45 ∼ 60 min to obtain single cells. After washed with PBS, cells were fixed with 4% paraformaldehyde (Yeasen, China) at room temperature for 20 min. Then the permeabilization was performed with 0.5% TritonX-100 for 10 min. Cells were analyzed by flow cytometer (BD, USA).

### RNA isolation and quantitative reverse transcription PCR (qRT-PCR)

The total RNAs of MaOs were extracted using the Total RNA Extraction Reagent (Yeasen, China). The Hifair^®^ III 1st Strand cDNA Synthesis SuperMix reagent kit (Yeasen, China) was used for cDNA synthesis. The 2×Hieff^®^PCR Master Mix (Yeasen, China) was used for PCR. The Hieff^®^ qPCR SYBR^®^ Green Master Mix (Yeasen, China) was used for RT-qPCR. The procedures were followed as the manufacturer’s instruction book. The primers were followed as below: *CK14* F: AGCTCAGCATGAAAGCGTCC, *CK14* R: TGGTCTTGTACTGGGTCAGG, 252 bp; *CK18* F: CAGATCGAGGAGAGCACCAC, *CK18* R: TCAGGCTGTTCTCCAAGCTG, 154 bp; *CK7* F GAGCCGTGAATATCTCTGTGGTC, *CK7* R TGTGGTCCTCATGGAGTAGG, 143 bp; *XDH* F: AGAAAGACCACAGGCAGGTTAC, *XDH* R: TGGAACGTCTTTCAGCCTCA, 122 bp; *FABP3* F: TGGCCAATATGACCAAGCCT, *FABP3* R: GTGTCACGATGGACTTGACCT, 165 bp; *SREBP1* F: CTGCTGACCGACATAGAAGACAT, *SREBP3* R: GTAGGGCGGGTCAAACAGG, 81 bp; *GLUT1* F: ATCCTCATCGCCCAGGTGTT, *GLUT1* R: GGTTCTCCTCGTTGCGGTTA, 166 bp; *GLUT4* F: TCAGGCATCAATGCGGTTTTC, *GLUT4* R: GAAGACTGTGTTGACCACGC, 108 bp; *CSN2* F: GGGAGTCCCCAAAGTGAAGG, *CSN2* R: CAAGACTGGACCAGAGGCAG 150 bp; *EIF4E* F: TACTCCAAATCCCCCGCCTA, *EIF4E* R: ACCAGAGTGCCCATCTGTTC, 100 bp; *GAPDH* F: TCCGTTGTGGATCTGACCTG, *GAPDH* R: CCCTGTTGCTGTAGCCGAAT, 250 bp.

The *GAPDH* was used as the reference gene. The annealing temperature of all PCR procedures was 55℃. The relative expression level of each gene was calculated using the 2^−ΔΔCt^ method.

### Immunofluorescence

The cultured MaOs of D21 were used for the expressions analysis of CK18 and CK14 through the immunofluorescence. Briefly, MaOs were fixed with 4% paraformaldehyde (Yeasen, China) at room temperature for 30 min. Then the permeabilization was performed with 0.1% TritonX-100 and blocked with 5% goat FBS for 1 h at room temperature. Then incubated with CK18 (Mouse anti-human, Solarbio, Cat: K200044M, China) and CK14 (Rabbit anti-human, Solarbio, Cat: K008794P, China) primary antibodies (1:500 in blocking solution) overnight at 4 °C. After incubated with Secondary IgG-FITC and IgG-RBITC antibodies (Solarbio, China) (1:500 in blocking solution) at 37℃ for 1 h, the nucleus was stained with DAPI (Solarbio, China). The Nikon AIR confocal microscope was used to imaging.

### Supernatants triglyceride (TG), lactose and CSN2 assay

At Day 4, all cultured MaOs were changed with fresh mediums. At Day 7, the supernatants were collected. The contents of TG, lactose and CSN2 were analyzed using Lactose Assay Kit, TG Assay Kit (Grace Biotechnology, China) and CSN2 Assay Kit (Abebio, China) and followed as Wang et al. [13].

Briefly, 20 µL and 10 µL supernatants were collected for TG and lactose assay. After incubated with specific reagents, the absorbance was read at 510 nm wavelengths using an Varioskan™ LUX Microplate Spectrophotometer (Thermo, USA). The concentrations were calculated based on standard curve equations.

100 µL supernatants were collected for CSN2 assay. After incubated at 37℃ for 2 h, the wells were washed 3 times. Then 100 µL/well Biotin-Conjugate (1×) was added and incubated at 37℃ for 1 h. After washed for 3 times, the Streptavidin-HRP reagent was added and incubated at 37℃ for 1 h. After washed for 5 times, the Substrate Solution was added and incubated at 37℃ for 15 ∼ 20 min. And after washed, 50 µL/well Stop Solution was added. The absorbance was read at 450 nm wavelengths using the Varioskan™ LUX Microplate Spectrophotometer (Thermo, USA) and the concentrations were calculated based on the standard curve equation.

At the same time, the concentrations of TG, lactose and CSN2 of fresh mediums were assayed for eliminate the background expression.

### Electroporation

The NEPA21 Electroporation system (Nepagene, Japan) was used for the MaOs transfection. The well-growed MaOs were selected and washed for 2 ∼ 3 times with pre-warmed PBS. Then gels were replaced into EP tubes and pre-chilled PBS was added to solubilization. Then centrifuged at 1200 PRM for 5 min and washed for 2 ∼ 3 times. The cleaned precipitation was digested with 0.25% trypsin for 3 ∼ 5 min. Then washed with PBS and centrifuged. The digested precipitation was resuspended with OPTI-MEM (10000/mL) and mixed with plasmid DNA (pCMV-GFP, Addgene #11153, 100 µg/mL). After incubated for 5 min at room temperature, the suspension was replaced into electric cup. The transmembrane voltage was set as 100, 150 and 200 V. After the transfection finished, the transfected MaOs were collected with centrifugation and plated as before. The fluorescence signal was detected after 48 h of transfection.

### Statistical analysis

The data are indicated as the means ± SE (*n* = 3). The significance was calculated using One-way ANOVA analyses in GraphPad Prism 8.0 statistical software (*n* = 3).

## Results

### Ovine MaOs isolation result with different digestion conditions

**Fig. 1:**
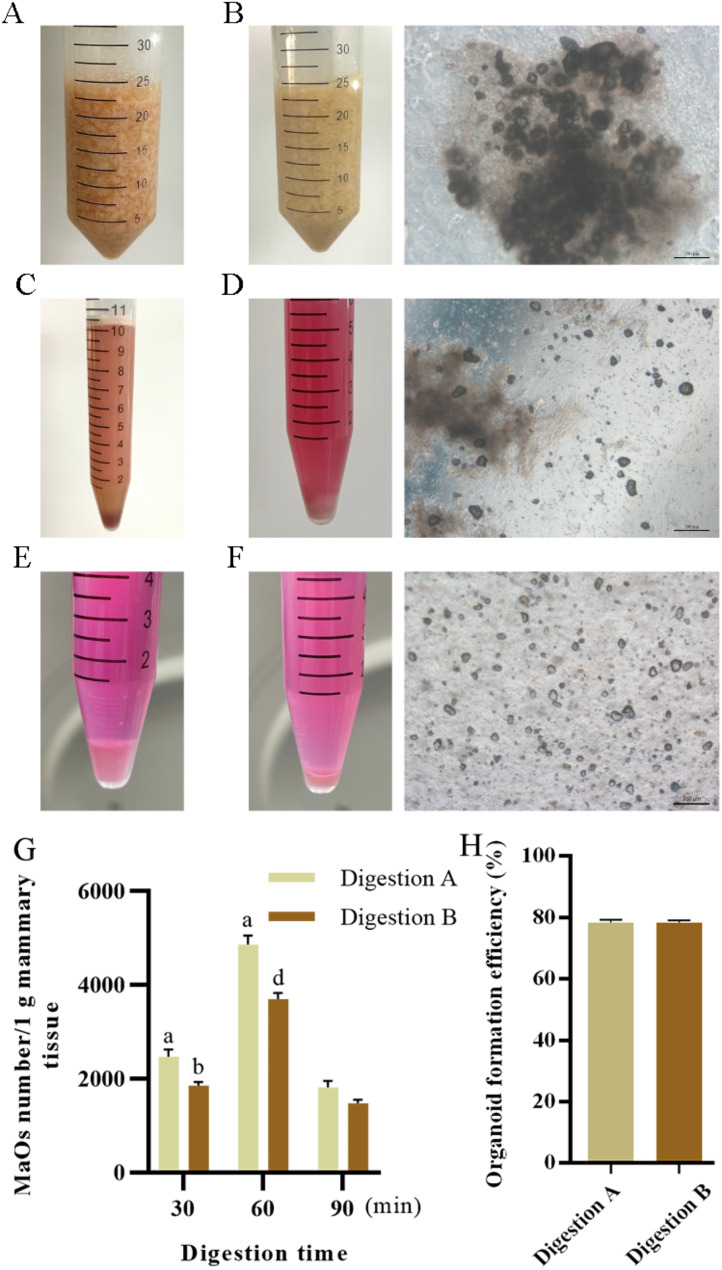
Isolation of mammary organoids (MaOs) from mammary tissue. (**A**) Image of undigested mammary tissue. (**B**) Mammary tissue after enzymatic digestion. (**C**) Digested tissue following centrifugation. (**D**) The fat layer was removed, and the pellet was resuspended. (**E**) Digested tissue fragments treated with DNase I. (**F**) Sedimentation of organoids after differential centrifugation. (**G**) Quantification of mammary organoid yield under different digestion conditions. (**H**) Organoid formation efficiency under different digestion methods. Adjacent letters (e.g., ‘a’ vs. ‘b’) represent *P* < 0.05, interspaced letters (e.g., ‘a’ vs. ‘d’) represent *P* < 0.001. (one-way ANOVA). Data are presented as mean ± SE (*n* = 3)

The fresh ovine mammary tissue was cut into pieces and the digestive enzyme solution was added resulting in a light pink mixture (Fig. 1A). After the digestion finished, the solution converted to milk white and some MaOs were mixed in tissue fragments (Fig. 1B). The digestive tissue fragments were collected with the cell sieve filtration. After centrifugation, the tissue fragments were at the bottom of tube (Fig. 1C). After the precipitates were resuspended, MaOs were visible (Fig. 1D). The precipitates were further digested with Dnase I and most fragments were eliminated (Fig. 1E). After 2 ∼ 3 washes, single cells have been eliminated largely and most of precipitates were MaOs (Fig. 1F).

Results showed that compared with the Digestion B, a greater number of mammary organoids were isolated by digestion with theDigestion A for 60 min (Fig. 1G). There was no significant difference of the organoid formation efficiency between two groups at D3, of which were between 76 ∼ 81% (Fig. 1H).

### Ovine MaOs culture optimization

**Fig. 2:**
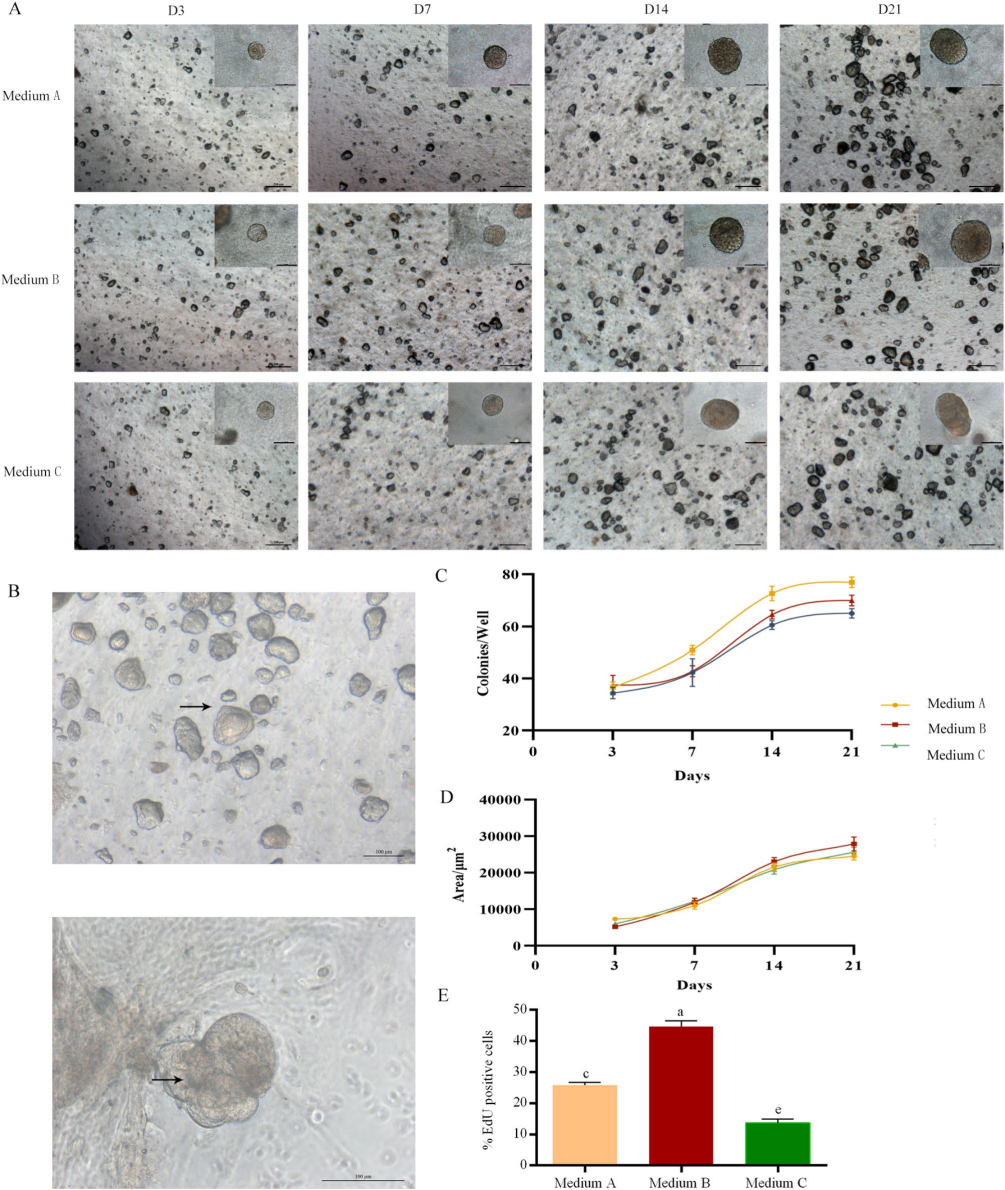
Morphology and proliferation capacity of cultured MaOs in Different Media. (**A**) Representative bright-field images of MaOs at days 3 (D3), 7 (D7), 14 (D14), and 21 (D21). Scale bars = 100 μm. (**B**) Physiological budding structures of MaOs. Upper panel: 100× magnification; lower panel: 200× magnification. (**C**) Growth curve showing the number of MaOs over 21 days. (**D**) Average area of individual organoids during 21-day culture. (**E**) Percentage of EdU-positive proliferating cells at D7. Interspaced letters (e.g., ‘a’ vs. ‘c’) represent *P* < 0.01. Data are presented as mean ± SE (*n* = 3)

This study found that with the increase of culture time, the edge color of MaOs became dark and gradually became smooth, and the central cavity became transparent (Fig. 2A). At D3 and D7, the MaOs in all three medium showed cystic cavity structure. However, the boundary of some MaOs was not obvious at D14 and D21 and presented with solid spheres with adhesion phenomenon in the medium C. On the contrary, the better growth status was observed in medium A and B. In addition, the physiological budding structures were observed in the medium B at D7 (Fig. 2B).

As shown in the Fig. 2C, the growth trends of MaOs in three medium were similar: the proliferation rate was slow before D7, then was increased and the plateau period was reached at D14. A higher density was observed in the medium B. The area analyze result showed that areas were similar in three medium at D3 and D7, while the medium B showed biggest at D14 and D21 (Fig. 2D). Furthermore, the percentage of proliferating cells in MaOs was assessed using EdU at D7. Results showed that MaOs in medium B had 44.60 ± 1.85% proliferating cells, extremely significantly higher than medium A (25.77 ± 0.90%, *P* < 0.001) and C (13.67 ± 1.25%, *P* < 0.001) (Fig. 2E, Fig S1). In summary, the medium B presented better growth effect for ovine MaOs.

### Cell types and lactation functional characterization of cultured ovine MaOs

**Fig. 3:**
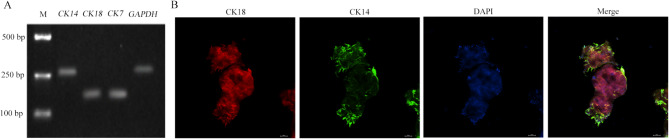
Cell types identification of cultured MaOs. Identification of Cell Types in Cultured MaOs. (**A**) Detection of cell type-specific mRNA (*CK14*, a marker for myoepithelial cells; *CK18* and *CK7*, markers for epithelial cells; and *GAPDH*, a housekeeping gene) in cultured MaOs using RT-PCR followed by ethidium bromide-stained agarose gel electrophoresis. (**B**) Immunofluorescence staining for CK18 (red) and CK14 (green), illustrating the presence of luminal and basal/myoepithelial cells, respectively. Nuclei are stained with DAPI (blue). Scale bars = 100 μm

To identify the cell types of cultured MaOs, the luminal epithelial cell markers of *CK7* and *CK18* and myoepithelial cell marker of *CK14* were analyzed through RT-PCR. The result showed that both of them were expressed (Fig. 3A). Furthermore, the immunofluorescence analyze showed that CK18 and CK14 were expressed in separate cells, of which CK18^+^ cells were located at the inner side and CK14^+^ cells were located at the outer side (Fig. 3B).

**Fig. 4:**
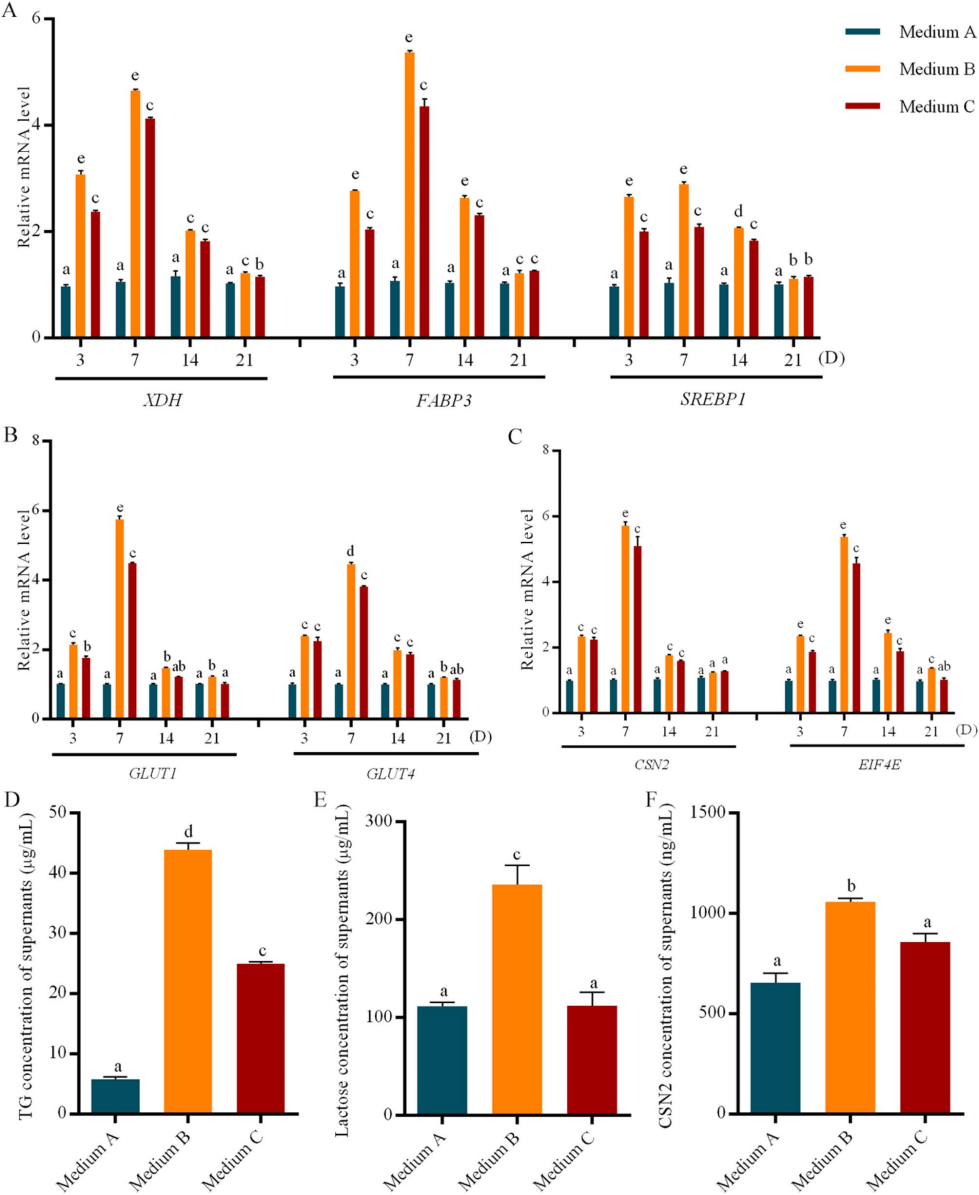
Expression of Milk Synthesis-Related Genes by MaOs cultured in medium A,** B and C.** (**A**) RT-qPCR analysis of genes involved in milk lipid metabolism (*XDH*, *FABP3* and *SREBP1*). (**B**) RT-qPCR analysis of genes associated with lactose metabolism (*GLUT1* and *GLUT5*). (**C**) RT-qPCR analysis of genes related to milk protein synthesis (*CSN2* and *EIF4E*). Data are relative to Medium A and are normalised to *GAPDH* (Housekeeping gene). (**D**) Triglyceride (TG) concentration in supernatants at D7. (**E**) Lactose concentration in supernatants at D7. (**F**) CSN2 concentration in supernatants at D7. Adjacent letters (e.g., ‘a’ vs. ‘b’) represent *P* < 0.05, interspaced letters (e.g., ‘a’ vs. ‘c’) represent *P* < 0.01, interspaced letters (e.g., ‘a’ vs. ‘d’) represent *P* < 0.001. Groups sharing at least one common letter are not significantly different. Data are presented as mean ± SE (*n* = 3)

To evaluate the lactational function of cultured Ovine MaOs, the expressions of milk lipid metabolism-related genes (*XDH*, *FABP3* and *SREBP1*), lactose metabolism-related genes (*GLUT1* and *GLUT4*) and milk protein synthesis-related genes (*EIF4E* and *CSN2*) were analyzed through qRT-PCR. Firstly, the gene expressions at D3, D7, D14 and D21 with different mediums were assessed. Results showed that for milk lipid metabolism-related genes, the expression of *XDH* in the medium B was higher than other two mediums at all time. And the expressions of *FABP3* and *SREBP1* in the medium B were also higher than other two mediums before D14, while showed the similar level with the medium C at D21 (Fig. 4A). For the lactose metabolism-related genes *GLUT1* and *GLUT4* and milk protein synthesis-related genes *EIF4E* and *CSN2*, expressions in the medium B were all higher than other two mediums at all time (Fig. 4B, C).

To analysis the secretory characteristics of cultured ovine MaOs, the supernatants at D7 were collected and concentrations of TG, lactose and CSN2 were assayed. Results showed that concentrations of TG, lactose and CSN2 in the medium B were significantly higher than medium A and C (*P* < 0.05) (Fig. 4D-F).

**Fig. 5:**
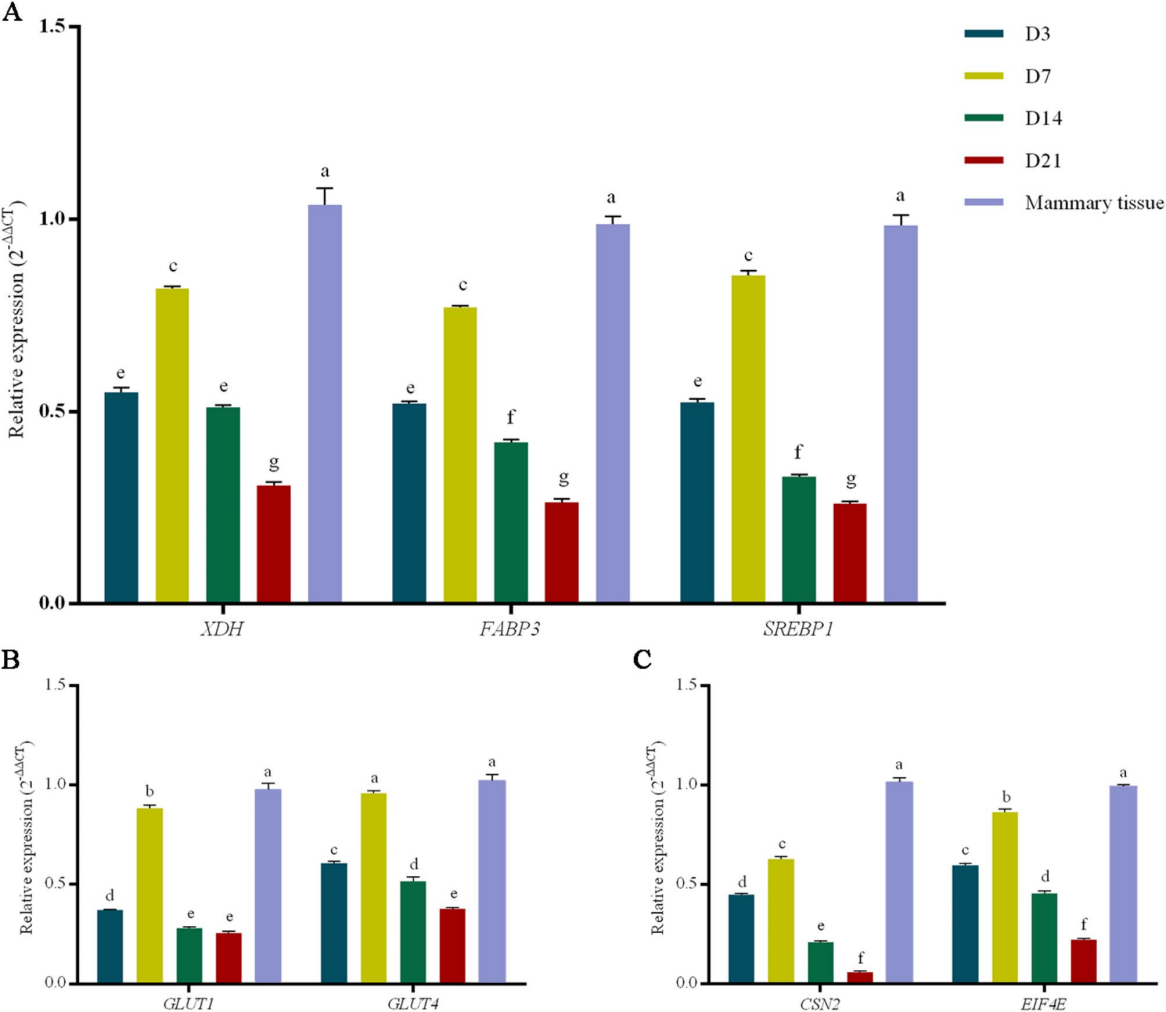
Expression of Milk Synthesis-Related Genes by MaOs cultured in Medium B over time. (**A**) RT-qPCR analysis of genes related to milk lipid metabolism (*XDH*, *FABP3* and *SREBP*) in mammary tissue and MaOs cultured in Medium B for 3, 7, 14 and 21 days. (**B**) RT-qPCR analysis of genes associated with lactose metabolism (*GLUT1* and *GLUT5*) in mammary tissue and MaOs cultured in Medium B for 3, 7, 14 and 21 days. (**C**) RT-qPCR analysis of genes related to milk protein synthesis (*CSN2* and *EIF4E*) in mammary tissue and MaOs cultured in Medium B for 3, 7, 14 and 21 days. Data are relative to mammary tissue and are normalised to *GAPDH* (Housekeeping gene). Adjacent letters (e.g., ‘a’ vs. ‘b’) represent *P* < 0.05, interspaced letters (e.g., ‘a’ vs. ‘c’) represent *P* < 0.01, interspaced letters (e.g., ‘a’ vs. ‘d’) represent *P* < 0.001. Data are presented as mean ± SE (*n* = 3)

Then the expression patterns of milk synthesis-related genes over time of MaOs in the medium B were analyzed. As shown in the Fig. 5, the highest expression was presented at D7 and then decreased. However, all of expressions of analyzed gene were lower than mammary tissue.

### Ovine MaOs transfection result

**Fig. 6:**
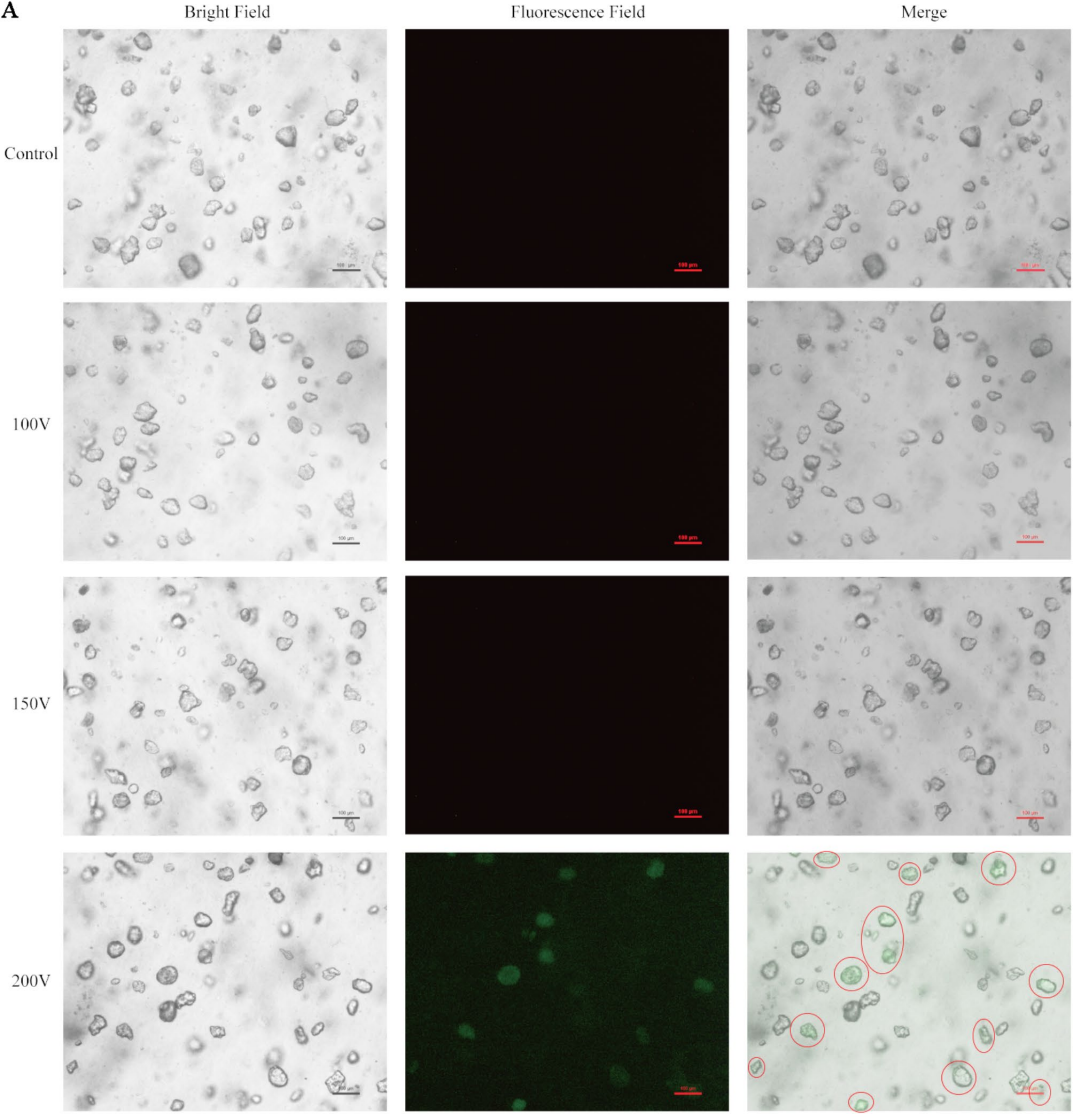
Electroporation and Transfection Efficiency in Cultured MaOs. (**A**) Representative images showing fluorescence signal from MaOs transfected with empty plasmid DNA with GFP at 48 h post-electroporation under different electroporation conditions (100 V, 150 V, 200 V). The red circles indicate that GFP^+^ MaOs. Scale bars = 100 μm **Supplementary**

In order to use cultured MaOs for further application, the transfection condition was explored. In this study, the electroporation voltages were set as 100, 150 and 200 V. As shown in the Fig. 6, only the 200 V group presented GFP^+^, about 27.3% MaOs were successfully transfected.

## Discussion

The organoid is one of ideal in vitro models for biological research. It owns advantages of similar to physiological cells composition and behavior, stable genome, suitable for high-throughput screening. In this study, the conditions of primary isolation, culturing, identification and transfection of ovine MaOs were systematically studied. The results provide a new method for the analysis of the ovine lactation traits regulatory mechanism.

In this research, we found that the type IV collagenase with the digestion for 60 min had higher ovine MaOs separation efficiency. The type IV collagenase is an important member of the matrix metalloproteinase (MMPs) family and can digest various tissues [14]. In addition, the digestion time is critical. If the time is too short, the tissue can not digest completely, while most of the tissue has been digested to single cells if the time is too long. Both can lead to a decreased efficiency of the organoid acquisition.

The medium of this study contains FGF2, EGF, PRL, L-Glu, ITS, HCT and heparin. The expression of EGF increases dramatically at the beginning of lactation, which indicates that the EGF plays an important role in the process of lactation [15]. The FGF signaling is essential for the mammary gland development and modulates the development of branching organs such as the lungs, salivary glands, kidneys, and mammary glands of mammals [16]. The FGF2 induces the fibroblast migration in the 3D extracellular matrix (ECM) by the actin cytoskeleton regulation and promotes forced-mediated collagen remodeling of breast fibroblasts. In addition, the FGF2 regulates the expression of ECM proteins in breast fibroblasts, including collagen, fibronectin, osteocyte protein, and MMPs [17]. The insulin promotes glucose and amino acid uptake, adipogenesis, intracellular transport, and synthesis of proteins and nucleic acids. The transferrin is a siderophore that helps to reduce the toxic levels of oxygen radicals and peroxides. The selenite is a cofactor and is used as an antioxidant in medium. The sodium pyruvate is a precursor of amino acids, fatty acids and cholesterol. It is an important intermediate in many biosynthetic pathways. All of these factors help to support the growth and physiological functions of cultured MaOs.

The identification of cell types is critical to assess the characteristic of organoids. It is the basic of functional mimic to the in vivo tissue. The mammary duct contains two layers that were composed of luminal and myoepithelial cells: inner (luminal) epithelial cells that produce milk, and outer (myoepithelial) cells that were contracted in response to the PRL during lactation to facilitate the milk discharge [18]. The myoepithelial cells are contacted with the basement membrane and are required to produce many basement membrane components, including laminin [19]. Furthermore, the CK18 and CK14 expressions at the protein level were verified. CK18, CK7 and CK14 are epithelial cells marker. The CK18 is luminal cells marker and the CK14 is base cells marker [20]. In this study, the *CK18*, *CK7* and *CK14* were expressed in cultured MaOs at the mRNA level. It suggests that the cultured ovine MaOs contain both luminal epithelial and myoepithelial cells. The luminal epithelial cells accounted for a large proportion, suggesting that the MaOs had lactational function. To assess the biological function of cultured MaOs, the expression of milk synthesis-related genes were analyzed. The milk fat synthesis is a complex molecular regulatory network that includes de novo synthesis of fatty acids, absorption of saturated long-chain fatty acids from blood, transport and desaturation of saturated long-chain fatty acids, synthesis of TG and secretion of lipid droplets [21]. The milk lactose exists in the form of free or oligosaccharides. It is synthesized and secreted by mammary epithelial cells and its concentration is closely related to the milk production [22]. The lactose is produced only in the Golgi apparatus of mammary epithelial cells. The extracellular glucose is taken up and then transported to the Golgi by the GLUT1 [23]. The mammalian milk protein synthesis is mainly regulated by the mTOR pathway. The EIF4E is a corn transcription factor of mTOR pathway [24]. In this study, the expressions of milk synthesis-related genes of the medium B were higher than other two. Compared with the medium A, the PRL was added in the medium B. It is helpful for the development of mammary gland and lactation. The PRL plays a role in the maintaining of Estrogen and Progesterone expressions and induces the mammary morphogenesis. If the PRL concentration is low in pregnant and lactation mice, the milk protein and milk production are seriously reduced. Our result was consistent with previous reports. In the medium C, the BSA was replaced with FBS and the adherent growth was observed. The function of lactation was impaired. Furthermore, the expressions of milk synthesis-related genes were highest at D7 and lowest at D21. At D3, the low expressions may be due to the fact that the MaOs has just been separated from the internal environment and has not yet adapted to the new environment. With the increasing of the culture time, the MaOs gradually adapt to the environment of matrix gel and utilize the growth factors in the medium. Results indicate that cultured MaOs at D7 is appropriate to further study.

The cell transfection can be summarized to three strategies: physical transfection, chemical transfection and biological transfection. The physical transfection contains electroporation, laser induction and particle bombardment. The chemical transfection mainly is cationic lipid transfection. And the biological transfection mainly uses viral vector. Previous, the transfections with adenovirus and lipofectin strategies were tried. However, none of them succeeded. It may be due to MaOs were cultured in the matrigel and with tight cell junction, the cationic lipid and viral vector are difficult to cross multiple layers of cells. Even the electroporation, suitable digestion is needed.

## Conclusion

This study successfully established the culture system of ovine MaOs. The cultured MaOs contain luminal and myoepithelial cells and presented lactational function. The electroporation was effective for the transfection of ovine MaOs.

In the further, the transcriptome analysis of cultured MaOs is needed to comprehensive evaluate its gene expression pattern. And the mammary stem cells derived MaOs culture system needs further study.

## Electronic supplementary material

Below is the link to the electronic supplementary material.

Results of EdU-positive cells assay at D7 by flow cytometry

Supplementary Material 2

## Data Availability

Te original contributions presented in the study are included in the article materials, further inquiries can be directed to the corresponding author.
