# Supplementary figures and images for: Establishment and characterization study of ovine mammary organoids
Source: BMC Vet Res. 2025 Mar 19;21:184. doi: 10.1186/s12917-025-04657-4 (PMC11921478; doi:10.1186/s12917-025-04657-4)

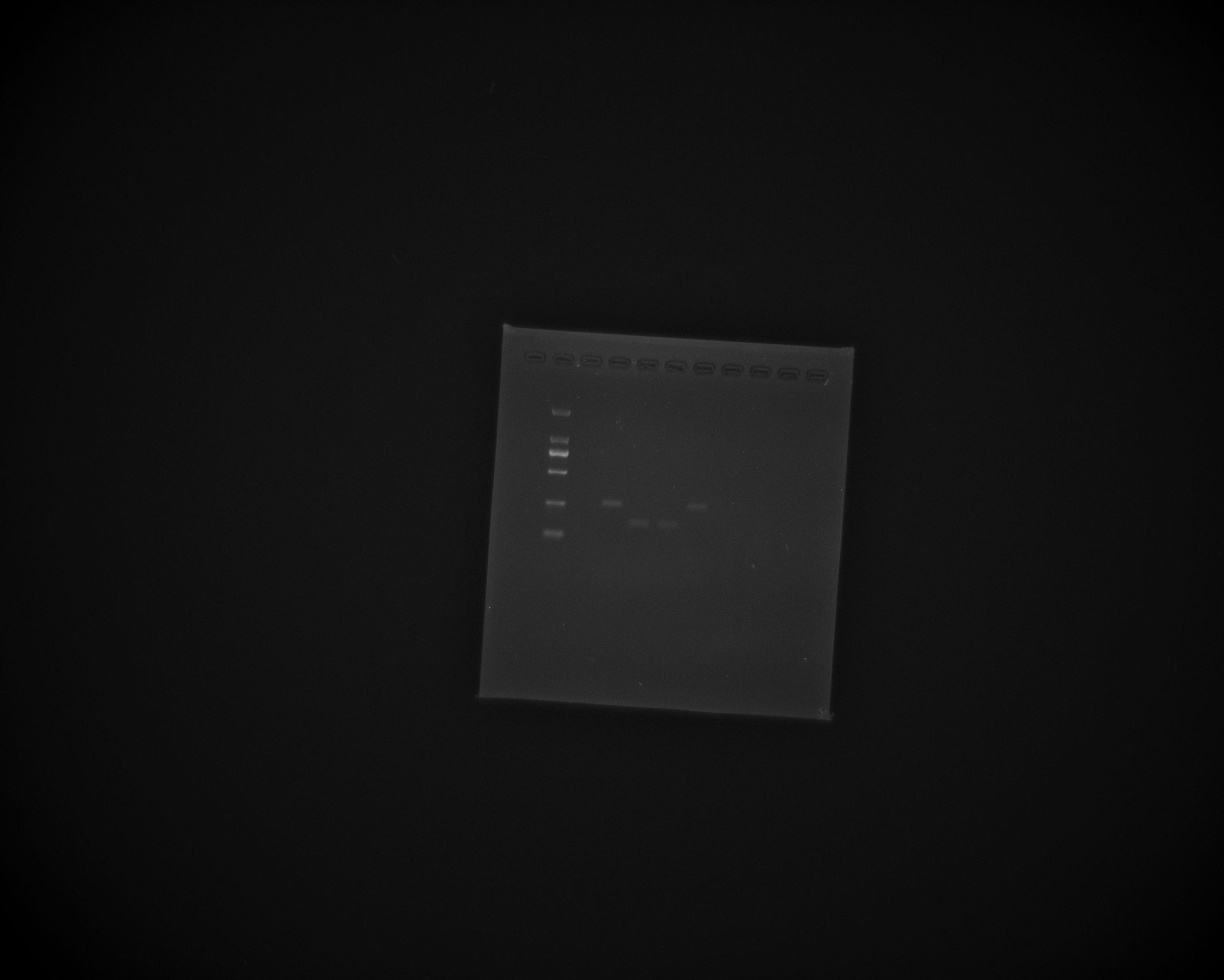

Supplement: Supplementary file 1 — Results of EdU-positive cells assay at D7 by flow cytometry [file 12917_2025_4657_MOESM1_ESM.png]

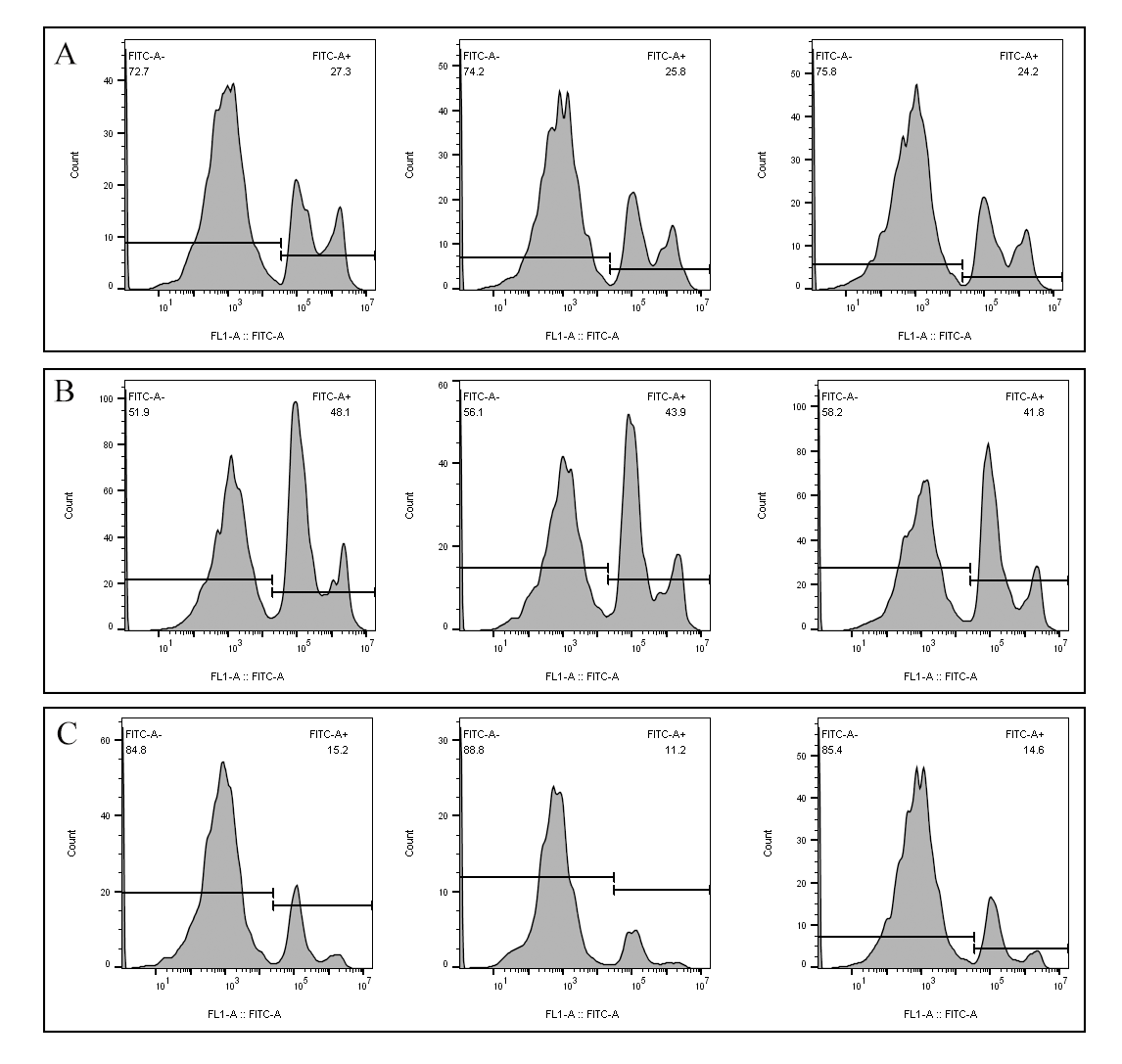

Supplement: Supplementary file 2 — Supplementary Material 2 [file 12917_2025_4657_MOESM2_ESM.png]
